# An Experimental Study of the Effect of Fancy Rope-Skipping on the Attention of Children

**DOI:** 10.3390/bs15121674

**Published:** 2025-12-03

**Authors:** Pan Li, Yanan Li, Yan Duan, Yaogang Han, Ying Liu

**Affiliations:** 1 School of Athletic Performance, Shanghai University of Sport, Shanghai 200438, China; 2 School of Psychology, Shanghai University of Sport, Shanghai 200438, China; 3 School of Psychology, Capital Normal University, Beijing 100048, China; 4 School of Physical Education, Shanghai University of Sport, Shanghai 200438, China; 5 Key Laboratory of Motor Cognitive Assessment and Regulation, Shanghai University of Sport, Shanghai 200438, China

**Keywords:** fancy rope-skipping, attention, graphic discrimination test, attention allocation

## Abstract

Introduction: Fancy rope-skipping is an emerging physical activity with numerous psychosocial benefits. However, its specific advantages for cognitive functions like attention, compared to traditional physical education, remain underexplored. Objective: This study aimed to investigate the effect of a 12-week fancy rope-skipping intervention on various domains of attention in fifth-grade students, compared to a standard physical education program. We hypothesized that the fancy rope-skipping intervention would yield significant improvements in one or more domains of attention (allocation, span, stability, and shifting). Methods: Two classes were randomly assigned as an experimental group (n = 28), which underwent a fancy rope-skipping program, and a control group (n = 31), which followed the regular syllabus. Attention was assessed using the Attention Quality Test before and after the intervention. Results: After controlling for pre-test scores, analysis of covariance revealed that the experimental group performed significantly better on the Graphic Discrimination Test, which measures attention allocation (F_(1,46)_ = 9.184, *p* = 0.004). No significant between-group differences were found in attention span, stability, or shifting. Conclusions: Fancy rope-skipping can specifically improve the ability to allocate attention in primary school students, highlighting its potential as a targeted intervention within physical education.

## 1. Introduction

As a newly emerging physical exercise, fancy rope-skipping—which combines dance, aerobics, music, and other fashionable elements—has been widely adopted in primary and secondary school physical education due to its low cost, ease of operation, fun, and safety, all of which significantly increase students’ interest in participation (J. Li, 2021). Furthermore, its unique portability allows students to exercise flexibly at different times and in various places.

As such, fancy rope-skipping serves as an effective means to enhance students’ physical and mental health. Beyond its well-documented physical health benefits, fancy rope-skipping demonstrates positive impacts spanning motor, psychological, and social domains, as supported by empirical data. Firstly, Liu Dong’ao confirmed that it significantly boosts the overall physical fitness of elementary students, laying a crucial corporeal foundation (Liu, 2016). Building on this, Lian Zhiying found that it effectively improved motor coordination in pupils, an essential individual skill (Lian, 2017). Extending to psychological well-being, Zhong Duo showed participation led to notably reduced anxiety and improved mental health in a university cohort (Zhong, 2015). Furthermore, at the social level, Li Bo et al. demonstrated that a structured program significantly enhanced adolescents’ interpersonal communication skills and reduced social distress, underscoring its role in fostering social adaptation (B. Li et al., 2017)

Despite these established benefits across motor, psychological, and social domains, a significant knowledge gap remains regarding its impact on cognitive functions. Fancy rope-skipping, as an emerging physical activity, has received a certain degree of research attention in terms of enhancing students’ physical fitness, mental health, and exercise motivation. However, there is a relative lack of research on how this activity affects cognitive functions, especially attention, and the existing literature has only explored the selective characteristics of attention, i.e., the ability to point to and focus on information, which is important for blocking out irrelevant interference and improving the efficiency of information processing, and which is considered to be a key cognitive structure connecting the dynamic interactions of perception, thinking, and behavior (Chen, 2004). Existing studies, however, have not thoroughly investigated other dimensions of attention, such as allocation, span, stability, and shifting.

Since academic performance is an important indicator of student learning effectiveness, cognitive functions such as attention are closely related to it (Shen, 1999). Besides, school-age children’s attention is in a rapid developmental stage and has a high degree of plasticity (Zhang, 2014). Therefore, improving these cognitive functions in students is of great importance. Acute fitness exercise not only promotes cognitive development and changes in neural potentials in the brain, but also has a positive effect on specific academic performance (Zhang, 2014). Long-term participation in fitness exercise, aerobic exercise, and physical fitness also promotes cognitive development and is positively correlated with academic performance, especially in executive control functions, where neural potential changes induced by prolonged exercise are more pronounced (Zhang, 2014). Moderate-intensity exercise programs, such as table tennis, children’s fitness boxing, and martial arts pile training, also had a positive effect on improving students’ attentional stability (Kong, 2013; Xu & Xu, 2013). Sustained physical activity, especially synchronized rope-skipping, has also been shown to be effective in improving students’ long-term working memory capacity, attention, information processing, and inhibitory switching ability (Song, 2024).

A growing body of research has begun to examine the cognitive benefits of coordinative and rhythmic physical activities like fancy rope-skipping. For instance, a recent study by Yamamoto demonstrated that long-rope jumping, an activity sharing core elements with fancy rope-skipping, was associated with increased central noradrenergic activation and improved attention maintenance (Yamamoto, 2021). This suggests a potential neurophysiological mechanism through which rope-skipping could enhance cognitive regulation. Furthermore, combination exercises incorporating fancy rope-skipping have shown positive effects on children’s overall attentional quality (Ni, 2018). However, these studies often employ multi-component interventions, making it difficult to isolate the unique contribution of fancy rope-skipping itself. Moreover, while attention is a multi-faceted construct, many existing studies lack a comprehensive assessment across its different domains, such as allocation, span, stability, and shifting (Yin, 2003).

Furthermore, attention is recognized as a multi-faceted construct. This study focuses on four core dimensions as conceptualized in psychometric models: (a) attention allocation, which refers to the ability to distribute limited cognitive resources across multiple tasks or stimuli simultaneously, forming the basis for effective multi-tasking; (b) attention span, defined as the number of distinct objects one can apprehend in a single moment, reflecting the breadth of perceptual awareness; (c) attention stability, the capacity to maintain focus on a task over a sustained period, resisting distraction; and (d) attention shifting, the ability to flexibly and deliberately switch the focus of attention between different tasks or mental sets according to changing demands. These domains represent distinct yet complementary aspects of overall attentional function. A comprehensive investigation into the impact of fancy rope-skipping, therefore, necessitates an assessment that can dissociate these specific facets. To address these gaps, the present study aims to isolate the effect of fancy rope-skipping by employing the Attention Quality Test (Yin, 2003), which is specifically designed to measure these four discrete attentional domains.

Fancy rope-skipping is hypothesized to offer superior benefits for attentional function compared to traditional physical education, due to its unique multi-faceted cognitive demands. The activity inherently requires a high degree of body coordination and precise timing, placing simultaneous loads on distinct attentional domains. Participants must allocate resources to monitor the rope’s trajectory and their own limb movements; maintain cognitive stability to adhere to a rhythmic structure; and rapidly shift focus during complex sequences or when responding to partners in team settings. These specific demands are largely absent in the more varied but less integrated activities of a conventional Physical Education curriculum. Therefore, we specifically hypothesized that a 12-week fancy rope-skipping intervention would lead to significantly greater improvements, particularly in the domains of attention allocation and shifting, compared to a traditional Physical Education program. To test this hypothesis, the present study employed a quasi-experimental design to investigate the effect of a 12-week fancy rope-skipping intervention on various domains of attention (allocation, span, stability, and shifting) in fifth-grade students, compared to a standard physical education program.

## 2. Method

### 2.1. Research Design

This study employed a quasi-experimental design with a pre-test and post-test control group. This design was selected because random assignment of individual students was not feasible within the school structure; instead, intact classes were assigned to either the experimental or control condition.

### 2.2. Participants

The experimental and control groups were formed through a class-level randomization procedure. Specifically, two parallel classes from the fifth-grade at HQ Elementary School were randomly selected. Subsequently, one of these classes was randomly assigned to serve as the experimental group (n = 28, mean age 10.85 ± 0.60 years, 17 girls and 11 boys), while the other was assigned as the control group (n = 31, mean age 11.10 ± 0.47 years, 13 girls and 18 boys). This method was employed to minimize selection bias at the group level, as random assignment of individual students was not feasible within the intact classroom structure of the school. Both groups were taught by the same physical education teacher to control for potential teacher effects. One student in the experimental class and two students in the control class were left-handed, and the rest were right-handed. All had normal hearing, normal or corrected-to-normal vision, no physical illness, and no history of psychiatric illness. The subjects and their guardians signed an informed consent form before the experiment and received a physical reward (a jump rope) after the experiment. The experiment was approved by the Ethics Committee of Shanghai University of Sport.

The sample size was determined by the total number of eligible students in the two randomly selected parallel classes. A post hoc power analysis conducted using G*Power 3.1.9.7 indicated that the final sample provided adequate statistical power (>0.80) to detect the main effect observed in the study.

### 2.3. Measures and Instruments

Attention was assessed using the Attention Quality Test (Yin, 2003). This tool comprises four paper-and-pencil subtests designed to measure distinct domains of attention: allocation, span, stability, and shifting. A detailed description of each subtest, including its name, task description, measured parameter, time limit, and psychometric properties, is provided in Table 1. The test was administered in a group format, and the guidelines were explained in detail to all participants prior to the initiation of the various subtests. The Attention Quality Test has demonstrated robust psychometric properties in previous research. During its development and validation, the test was administered to a total of 656 students. Subsequent analyses, including factor analysis and correlation assessments, confirmed its sound construct and criterion-related validity. Factor analysis confirmed the test’s construct validity, establishing that its four subtests measure distinct attentional domains: allocation, span, stability, and shifting. This was further supported by an additional validation study involving 221 students and 95 adolescent athletes, which found that test scores showed significant convergence with concurrent ratings of attention provided by the students themselves, their parents, and their teachers. The test exhibited strong criterion-related validity, significantly discriminating between groups of adolescents identified as having high versus low attention levels (*p* < 0.05). These comprehensive studies collectively affirm the Attention Quality Test as a valid and appropriate instrument for the present research context.

### 2.4. Procedure

The study received full approval from the Ethics Committee of Shanghai University of Sport (No. 2014016). Prior to the study, written informed consent was obtained from all participants’ parents or guardians, and verbal assent was obtained from the children themselves. The entire experiment spanned 14 weeks: the pre-test of attention, the implementation of different physical education programs, and the attention post-test. The pre-test was administered one week prior to the official physical education session, and attention levels were measured in the classroom. The implementation of the different physical education programs was scheduled during physical education classes and was done on the playground, following the different physical education programs (regular instruction/specialized jump rope instruction) for 12 weeks of physical education. The post-test of the attention test was scheduled one week after the end of the sports intervention, with the same content as the pre-test.

### 2.5. Intervention

Experimental Group (Fancy Rope-Skipping): The intervention was a structured, 12-week program, conducted twice weekly. Each 40 min session was strictly structured: (a) a 5 min warm-up involving joint mobilization and light jogging; (b) a 30 min main session dedicated to progressive fancy rope-skipping drills; and (c) a 5 min cool-down with static stretching. The program was designed to progress according to the students’ level of acceptance, beginning with preparatory activities and basic individual patterns (e.g., twisted rope, interlocking ropes, double flying patterns, crotch jumps, salute jumps) and advancing to more complex interactive rope patterns (e.g., switching rope, netting, out of the person rope skills) and wheel jumping patterns. The program progressed weekly, systematically increasing the complexity of skills from basic swings and jumps to coordinated partner and group routines (for the detailed 12-week program, see Table 2). Crucially, exercise intensity was systematically monitored and controlled. Before each class, three students were randomly selected to wear a Polar heart rate monitor. This sampling method is a common and practical approach in school-based exercise intervention studies to minimize disruption while providing a valid estimate of group exercise intensity. It was assumed that as the entire class participated in the same structured, teacher-led activity, the exertion levels of randomly selected students would be representative of the group. The chest strap was secured comfortably, and the heart rate was recorded during the session. The target intensity was set at 70–90% of maximum heart rate (calculated as 220—age), corresponding to a range of 125–190 beats per minute, which was maintained for a total of 20 min during the core training portion to ensure a medium-high intensity level.

Control Group (Traditional Physical Education): The control group followed the standard physical education syllabus over the same 12-week period, adhering to the school’s regular curriculum. This ensured that both groups were matched for the overall duration and frequency of physical activity. Their classes also lasted 40 min, typically comprising a 5 min warm-up, a 30 min main training segment, and a 5 min relaxation period. The curriculum included a variety of standard activities such as running, ball games, and gymnastics (for the detailed weekly schedule, see Table 3). The key distinction was the content of the main session; the control group engaged in diverse traditional physical activities that lacked the specific coordinative, rhythmic, and cognitive demands inherent in the fancy rope-skipping protocol. To allow for a fair comparison, exercise intensity was likewise monitored in the control group using the same Polar heart rate system and protocol (targeting 125–190 bpm for 20 min), ensuring that any observed effects could be more confidently attributed to the type of activity rather than differences in physiological exertion.

### 2.6. Data Analysis

Data were excluded from the analysis for a specific subtest if a student was absent during that test session or if their answer sheet was returned incomplete (e.g., blank or with markings that clearly indicated a failure to understand or attempt the task. This resulted in varying sample sizes (n) across the four subtests, as detailed in Table 4. All statistical analyses were performed using SPSS Statistics (Version 22.0). Preliminary analyses included independent samples *t*-tests and chi-square (χ^2^) tests to ensure the baseline equivalence of the two groups on demographic and pre-test variables. The normality of the data distribution was verified to ensure the assumptions for parametric tests were met. The primary analysis to test the intervention effect on post-test scores was conducted using analysis of covariance (ANCOVA), with the pre-test score as the covariate. This was performed separately for each of the four attention subtests. The assumption of homogeneity of regression slopes was checked and met for all analyses. Statistical significance was set at *p* < 0.05.

## 3. Results

Preliminary independent samples *t*-tests and a chi-square test confirmed that the two groups were comparable at baseline in terms of age, body mass index (BMI), and gender distribution. Differences in age, body mass index (BMI) and gender composition between the two classes were not statistically significant (age: t_(57)_ = −1.73, *p* = 0.09; gender: χ^2^ = 2.08, *p* = 0.15; BMI: experimental class, 18.32 ± 4.60, control class: 17.87 ± 3.07, t_(57)_ = −0.45, *p* = 0.66).

Baseline equivalence on the attentional measures was also assessed. There was no statistically significant difference between the performance of the two groups of students on the three attention subtests (Test I, Test III, and Test IV) prior to the intervention (Graphic Discrimination Test (Test I, attention allocation): t_(47)_ = 1.37, *p* = 0.18; Visual Tracking Test (Test III, attention stabilization): t_(51)_ = 1.74, *p* = 0.09; and Addition and Subtraction Test (Test IV, attention shift): t_(44)_ = −0.94, *p* = 0.35). There was a statistically significant difference between the two groups of students in one of the tests, the Pick Four Circles Test (Test II, Attentional Span), even before the intervention, t_(54)_ = 2.33, *p* = 0.02. The raw pre-test scores and results of these baseline comparisons are detailed in Table 4. This baseline discrepancy poses a limitation to the internal validity of the study regarding the span domain, as it suggests that the two groups may not have been fully comparable in attentional span prior to the intervention. Although ANCOVA was later used to control for pre-test scores, the presence of a pre-existing group difference in one attentional component highlights the challenge of achieving full equivalence in quasi-experimental designs where random assignment at the individual level is not feasible. This finding underscores the importance of interpreting non-significant results in the span domain with caution, as the lack of post-intervention difference may be partially attributable to initial group disparity rather than a true null effect of the intervention. Future studies employing individual-level randomization or matched-pair designs would help mitigate such pre-test imbalances and allow for clearer causal inferences across all attentional domains.

**Figure 1:**
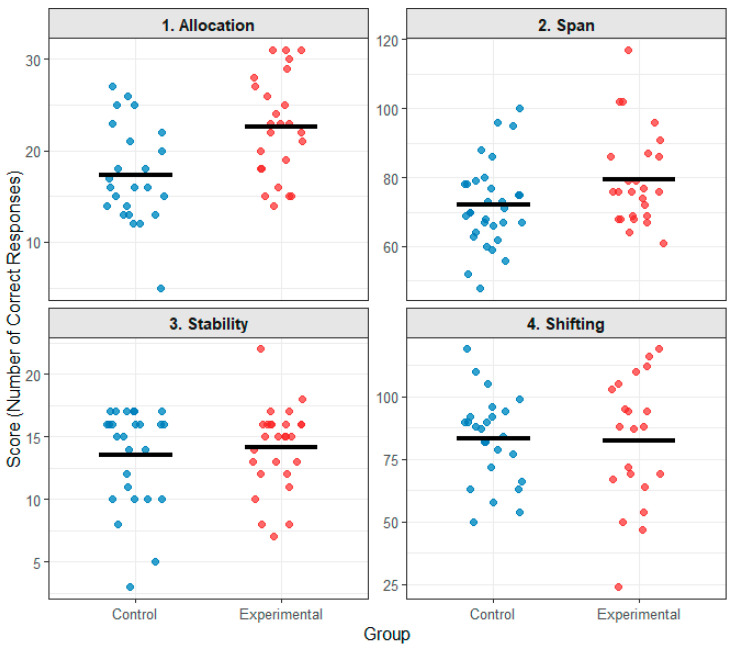
Raw post-test scores on the Attention Quality Test for the experimental and control groups. The plot displays individual participant scores (dots) and group means (horizontal bars) for the control and experimental groups in the allocation, span, stability, and shifting domains. The figure displays unadjusted descriptive data; the statistical analysis based on ANCOVA, which controls for pre-test scores, is presented in text.

There was a significant linear correlation between scores on the pre-test and the post-test (Pearson’s Linear Correlation, Graphic Discrimination Test (Test I, Attentional Distribution): r = 0.47, *p* = 0.001; Pick Four Circles Test (Test II, Attention Span): r = 0.74, *p* < 10^−10^; Visual Tracking Test (Test III, Attention Stabilization): r = 0.46, *p* = 0.001; Addition and Subtraction Test (Test IV, Attention Shifting): r = 0.79, *p* < 10^−10^). Therefore, in order to eliminate the effect of pre-test scores on post-test scores, post-test scores (the number of correct responses in the four tests) of the experimental and control groups in the current study were analyzed for covariance with pre-test scores as the covariate. The results revealed that after eliminating the effect of the pre-test, there was a statistically significant difference in performance between the experimental and control groups only on the graphic discrimination test (Test 1, Attention Distribution), F_(1,46)_ = 9.184, *p* = 0.004, partial η^2^ = 0.166. The adjusted mean for the experimental group was significantly higher than that of the control group (Experimental: 22.18 ± 0.99; Control: 17.81 ± 1.02). This significant improvement in attention allocation for the experimental group suggests that the complex sensorimotor coordination and rhythmic timing required for fancy rope-skipping may place unique demands on, and thereby enhance, the brain’s ability to efficiently distribute cognitive resources across multiple stimuli. There was no statistically significant difference in performance between the experimental and control groups on the other three tests (Test 2, Selection of the Four Circles Test, F_(1,53)_ = 0.427, *p* = 0.516, partial η^2^ = 0.008; Adjusted Means—Experimental: 76.25 ± 1.87, Control: 74.57 ± 1.67; Test III, Visual Tracking Test, F_(1,50)_ = 0.021, *p* = 0.886, partial η^2^ < 0.001; Adjusted Means—Experimental: 13.76 ± 0.64, Control: 13.90 ± 0.66; Test IV, Addition and Subtraction Test, F_(1,43)_ = 0.860, *p* = 0.35, partial η^2^ = 0.020; Adjusted Means—Experimental: 84.81 ± 2.91, Control: 81.12 ± 2.67). The raw post-test scores and full ANCOVA results for all subtests are available in Table 4. The pattern of unadjusted post-test scores across all domains for both groups is visually summarized in Figure 1. The absence of significant intervention effects on attention span, stability, and shifting indicates that the benefits of fancy rope-skipping might be domain-specific. It is plausible that these aspects of attention are either less malleable through this specific type of physical training or are engaged differently, potentially requiring a longer or more intensive intervention to manifest measurable changes.

## 4. Discussion

The results of the current study showed that fifth graders’ posttest scores on the Graphic Discrimination Test were significantly higher in the experimental class than in the control class after excluding interference from the pretest scores, i.e., fancy rope-skipping had a significant positive effect on improving fifth graders’ attention allocation.

Fancy rope-skipping, as a physical activity, has received attention from researchers in recent years, especially in terms of its effects on children’s attentional quality (B. Li et al., 2017; Lian, 2017; Liu, 2016; Zhong, 2015). Our finding that fancy rope-skipping specifically enhanced attention allocation aligns with, yet refines, previous literature. For instance, Ni Q found that a combined intervention of basketball and fancy rope-skipping improved overall attentional quality, with a reported large effect size (Ni, 2018). However, by employing a pure fancy rope-skipping intervention, our study allows us to attribute the observed benefit in attention allocation more directly to this specific activity, rather than to a combination of sports. The critical role of speed training in enhancing attention, as highlighted by Li et al., offers a compelling lens through which to view our own findings (S. Li et al., 2012). In the context of fancy rope-skipping, “speed” transcends mere physical velocity; it embodies the rapid processing of sensory information (rope position, rhythm) and the swift allocation of cognitive resources to plan and execute precise motor responses. Therefore, the speed quality inherent in fancy rope-skipping does not merely improve reaction time, but fundamentally trains the brain’s capacity for rapid and efficient attention allocation under time pressure. This provides a plausible mechanistic explanation for why our intervention specifically boosted performance on the allocation-oriented Graphic Discrimination Test.

However, this beneficial effect was domain-specific. Examination of the children’s performance on the other three subtests—namely, the Selective Circle Test (attention span), the Visual Tracking Test (attention stability), and the Addition and Subtraction Test (attention shifting)—revealed no statistically significant differences between the experimental and control groups. The fact that improvements were observed solely in attention allocation can be interpreted by the unique cognitive demands of fancy rope-skipping. Unlike sustained focus tasks (training stability) or task-switching paradigms (training shifting), fancy rope-skipping inherently constitutes a complex, multi-tasking activity. Participants must continuously allocate their attentional resources between multiple concurrent processes: tracking the rope’s path, timing their jump, coordinating limb movements, and often synchronizing with partners or music. This continuous requirement to manage multiple streams of information places a direct and high load on the brain’s attentional control networks responsible for resource allocation. This explanation is bolstered by the findings of Xiong and Tang (2015), who demonstrated the superiority of martial arts—another discipline requiring high body coordination and spatial awareness—in improving attentional allocation. Thus, the improvement observed in our study is likely not a general, non-specific effect of exercise, but rather a specific training effect on the neural circuits engaged by the coordinative and rhythmic challenges of the activity itself (Xiong & Tang, 2015). Furthermore, some studies have found that the mechanism behind this may be the existence of a significant effect of rope jumping on brain-derived monoamine neurotransmitters associated with cognitive regulation, with an increase in 3-methoxy-4-hydroxyphenylglycol associated with increased attention during long rope jumping. Researchers have noted that jumping long rope corresponds to central noradrenergic activation and related maintenance of attention (Yamamoto, 2021).

## 5. Limitations, Implications, and Future Directions

This study has several limitations that should be considered. The quasi-experimental design, while pragmatic, limits the random assignment of participants. The sample was also drawn from a single grade and school, which may affect the generalizability of the results. Furthermore, the reliance on behavioral measures alone means the underlying neurophysiological mechanisms remain inferred.

Notwithstanding these limitations, the findings hold clear practical significance. They provide physical educators and school administrators with empirical evidence that integrating fancy rope-skipping into the standard curriculum is a viable strategy for enhancing children’s attentional allocation—a critical cognitive skill for academic learning. As a low-cost, enjoyable, and safe activity, it represents a valuable tool for promoting cognitive development alongside physical health.

Future research should therefore build upon this foundation by employing randomized controlled trials with more diverse populations. A key priority is to incorporate neurophysiological measures (e.g., fNIRS, EEG) to directly probe the neural mechanisms underlying the observed behavioral improvements. Additionally, studies comparing fancy rope-skipping with other coordinative sports and exploring its effects across different age groups would help to further delineate its specific role in cognitive development.

## 6. Conclusions

This study demonstrates that a 12-week fancy rope-skipping intervention can specifically enhance the ability to allocate attention in fifth-grade students, compared to a traditional physical education program. This finding aligns with the study’s primary objective and underscores that the cognitive benefits of physical activity can be domain-specific, likely driven by the unique multi-tasking and coordinative demands of fancy rope-skipping, which place a direct load on attentional control networks.

## Figures and Tables

**Table 1:** Description and psychometric properties of the Attention Quality Test subtests.

Attentional Domain	Subtest Name	Task Description & Measured Parameter	Time Limit	Parameter	Reliability & Validity Evidence
Allocation	Graphic Discrimination Test	Identify and mark target graphics among distractors.	3 min	Number of correct responses	sound construct and criterion-related validity
Span	Selection of Four Circles Test	Quickly identify and select target circles.	3 min		
Stability	Visual Tracking Test	Track a visual path without distraction.	2 min		
Shifting	Addition and Subtraction Test	Alternate between addition and subtraction operations.	3 min		

**Table 2:** 12-Week fancy rope-skipping intervention program (experimental group).

Week	Skill-1	Schematic Diagram	Skill-2	Schematic Diagram
1	Side Swing	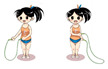	Basic Jump	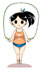
2	Side Swing Jump	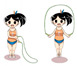	Under-leg A Jump	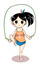
3	Jumping Jack	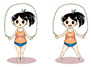	Two-in-One-Rope: Alternating Jump	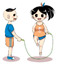
4	Under-leg B Jump	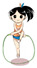	Two-in-One-Rope: Jumping Jack	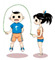
5	High Knee Jump	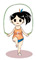	Two-in-One-Rope: Simultaneous Jump	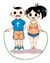
6	Kick Jump	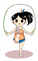	Single Rope with Partner	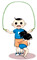
7	Turn Around Jump	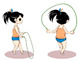	Net Rope Jump	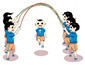
8	Cross-over Jump	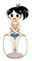	Two-in-One-Rope: Cross-over Jump	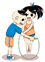
9	Double Under	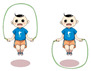	Double Dutch (Wheel Jump)	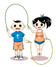
10	Double Under Variations	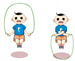	Double Dutch (Basic)	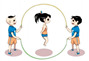
11	Fancy Move Combination (4 moves in series)		Double Dutch (Crossover)	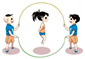
12	Fancy Move Combination (8 moves in series)		Rainbow Jump	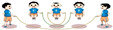

**Table 3:** Class schedule of regular teaching contents (control group).

Week	Teaching Contents
1	1. Rhythmic exercise: radio exercise 2. Formation: formation comprehensive exercise
	1. Rhythmic exercise: radio exercise 2. Formation: formation comprehensive exercise
2	1. Rolling: far support front roll 2. Climbing: climb over a mat of a certain height
	1. Rolling: far support front roll 2. Climbing: climb over a mat of a certain height
3	1. Walking and running: fast running 2. Small balls: small basketball
	1. Walking and running: fast running 2. Small balls: small basketball
4	1. Walking and running: fast running 2. Integrated activities: hopscotch
	1. Draping and bracing: bracing for movement 2. Integrated activities: climbing over obstacles for relay runs
5	1. Throwing: front solid ball throw 2. Folk sports: martial arts combination moves
	1. Throwing: front solid ball throw 2. Folk sports: martial arts combination moves
6	1. Throwing: front throw solid ball, test 2. Small ball: small basketball
	1. Jumping: jumping up into a kneeling support—kneeling and jumping down 2. Integrated activities: cockfighting
7	1. Jumping: jumping up into a kneeling brace-kneeling jump down 2. Folklore: 1 min short rope
	1. Jumping: jumping up into a kneeling brace-kneeling jump down 2. Folklore: 1 min short rope
8	Sports Basics: Nutrient-Dense Foods
	1. Walking & running: chase running 2. Small balls: small basketballs
9	1. Walking and running: timed round-trip running 2. Integrated activity: the eagle catches the chicken
	1. Small ball: soccer 2. Integrated activity: bouncing ball
10	1. Walking & running: timed round trip 2. Small balls: badminton
	1. Jumping: squatting, long jump 2. Folklore: 1 min short rope
11	1. Jumping: squatting, long jump 2. Folklore: 1 min short rope
	1. Jumping: squatting, long jump 2. Integrated activities: fried soybeans
12	1. Jumping: squatting, long jump 2. Integrated activities: pushing carts
	1. Jumping: squatting, long jump, assessment 2. Small ball: badminton

**Table 4:** Attention Quality Test raw scores (mean ± standard deviation) and inferential statistics for experimental and control classes.

Test (Attentional Domain)	Group	n	Pre-Test	Post-Test	Inferential Statistics (Group Comparison)
Test I: Graphics Discrimination	Experimental	25	15.44 ± 5.93	22.64 ± 5.52	Pre-test: t_(47)_ = 1.37, *p* = 0.18
(Allocation)	Control	24	13.29 ± 5.02	17.33 ± 5.38	Post-test (ANCOVA): F_(1,46)_ = 9.18, *p* = 0.004
Test II: Select Four Circles	Experimental	25	68.96 ± 14.13	79.44 ± 13.64	Pre-test: t_(54)_ = 2.33, *p* = 0.02
(Span)	Control	31	61.45 ± 9.98	72.00 ± 12.26	Post-test (ANCOVA): F_(1,53)_ = 0.43, *p* = 0.52
Test III: Visual Tracking	Experimental	27	14.70 ± 3.36	14.15 ± 3.32	Pre-test: t_(51)_ = 1.74, *p* = 0.09
(Stability)	Control	26	13.15 ± 3.12	13.50 ± 3.96	Post-test (ANCOVA): F_(1,50)_ = 0.02, *p* = 0.89
Test IV: Addition & Subtraction	Experimental	21	69.33 ± 18.82	82.24 ± 25.61	Pre-test: t_(44)_ = −0.94, *p* = 0.35
(Shifting)	Control	25	74.40 ± 17.77	83.28 ± 17.35	Post-test (ANCOVA): F_(1,43)_ = 0.86, *p* = 0.36

## Data Availability

The data that has been used is confidential. All data generated or analyzed during this study are available and can be provided if required.
